# Metabolomic Analysis of the Liver of a Dextran Sodium Sulfate-Induced Acute Colitis Mouse Model: Implications of the Gut–Liver Connection

**DOI:** 10.3390/cells9020341

**Published:** 2020-02-01

**Authors:** Sou Hyun Kim, Wonho Lee, Doyoung Kwon, Seunghyun Lee, Seung Won Son, Min-Soo Seo, Kil Soo Kim, Yun-Hee Lee, Suhkmann Kim, Young-Suk Jung

**Affiliations:** 1 Lab of Molecular Toxicology, College of Pharmacy, Pusan National University, Busan 46241, Korea; 2 Department of Chemistry, Center for Proteome Biophysics and Chemistry Institute for Functional Materials, Pusan National University, Busan 46241, Korea; 3 Department of Cellular and Molecular Pharmacology, University of California San Francisco, San Francisco, CA 94158-2280, USA; 4 Laboratory Animal Center, Daegu-Gyeongbuk Medical Innovation Foundation, Daegu 41061, Korea; 5 College of Veterinary Medicine, Kyungpook National University, Daegu 41566, Korea; 6 College of Pharmacy and Research Institute of Pharmaceutical Sciences, Seoul National University, Seoul, 08826, Korea

**Keywords:** metabolomics, colitis, gut and liver connection

## Abstract

The incidence of ulcerative colitis (UC) is increasing worldwide, and it has become a growing problem in Asia. Previous research on UC has focused on serum, plasma, urine, gut tissues, and fecal metabolic profiling, but a comprehensive investigation into the correlation between the severity of colitis and changes in liver metabolism is still lacking. Since the liver and gut exchange nutrients and metabolites through a complex network, intestinal diseases can affect both the liver and other organs. In the present study, concentration-dependent dextran sodium sulfate (DSS)-induced ulcerative colitis was employed to examine changes in liver metabolism using a proton nuclear magnetic resonance spectroscopy (^1^H-NMR)-and ultra-performance liquid chromatography time of flight mass spectroscopy (UPLC-TOF MS)-based metabolomics study. Using the multivariate statistical analysis method orthogonal projections to latent structures discriminant analysis (OPLS-DA), changes in metabolites depending on the DSS dose could be clearly distinguished. Specifically, hepatic metabolites involved in one-carbon metabolism, carnitine-related metabolism, and nucleotide synthesis were found to be affected by intestinal inflammation, implying the existence of a metabolic connection between the gut and liver. We are currently investigating the significance of this metabolic condition in UC.

## 1. Introduction

Ulcerative colitis (UC) is an inflammatory bowel disease (IBD), which includes chronic inflammation of the gastrointestinal tract [1]. The primary symptoms of UC are acute and chronic inflammation of the mucosa, diarrhea, and rectal bleeding [2]. The incidence of IBD is increasing in Asians because of the westernization of their lifestyle and changes in environmental factors [3]. Recent studies have suggested that the etiology of IBD is multifactorial, resulting from the interplay of immunological, molecular, genetic, microbial, diet, drug use-related, and environmental factors [1,4,5]. IBD is considered to affect liver homeostasis, and in fact, the occurrence of non-alcoholic fatty liver disease (NAFLD) has been found in IBD patients [6,7,8]. Anatomically, it is easy to suppose that abrogated homeostasis in the gut affects liver health. Obtaining about 70% of the blood supply from the portal vein, which is the direct venous outflow of the intestine, the liver is the first and main organ exposed to gut-derived substances, such as ingested nutrients and bacterial products [9]. Crohn’s disease (CD) patients, characterized by a loss of epithelial barrier, as well as chronic inflammation in the intestine, display enhanced bacterial colonization in portal blood and liver, further emphasizing the fact that disruption of the intestinal barrier increases the hepatic exposure of intestinal microbes [10]. 

Bacteria and their products can be harmful or beneficial to the liver, depending on the situation. Exposure to high levels of lipopolysaccharide (LPS) in the liver in certain disease states can lead to the recruitment of inflammatory cells, destroying the parenchyma of the liver [11]. Increased hepatic and blood LPS and peptidoglycans—bacterial products derived from the intestine due to an increased gut permeability—are found in alcoholic liver disease [12,13,14]. The activation of toll-like receptors (TLRs) specific for bacterial products in Kupffer cells and other liver cells promotes a series of responses resulting in the generation and release of inflammatory cytokines such as TNF-α and IL-1β. This leads to excessive inflammatory reactions and eventually damages liver cells [15]. It is well-known that an increase in intestinal microbial products causes or worsens liver disease. However, no pronounced inflammatory response was shown under constant exposure to low concentrations of LPS in the liver [16], suggesting that there is a threshold to activate the inflammatory response. On the contrary, it has also been shown that bacteria and their products have beneficial effects on the liver. Mice supplemented with broad-spectrum antibiotics to reduce intestinal microflora displayed an impairment of liver regeneration following partial hepatectomy [17]. Furthermore, a beneficial role of symbiotic microflora was demonstrated in models of alcoholic liver injury and hepatic fibrosis, which were protected by microbiota under certain circumstances [18,19]. Although the significance of the relationship between the intestinal tract and liver has been studied, extensive research is required to elucidate the interactions and their consequences in detail.

Recently, metabolite profiling of the blood, urine, gut tissues, and feces of mice and humans with IBD lesions has revealed unique characteristics that can be applied to diagnose IBD by distinguishing between IBD patients and healthy controls [20,21,22,23,24,25]. However, there is a lack of comprehensive studies investigating the correlation between the severity of colitis and changes in liver metabolism. In this study, we hypothesized that the degree of intestinal inflammation affects liver homeostasis, and investigated the relationship between the severity of colitis and changes in metabolites in the liver. To comprehensively profile metabolic changes in the liver, depending on the severity of colitis, we used proton nuclear magnetic resonance spectroscopy (^1^H-NMR) and ultra-performance liquid chromatography time of flight mass spectroscopy (UPLC-TOF MS). Subsequently, the multivariate statistical method [26] and target profiling method [27] were applied to analyze the acquired data and search for metabolites characteristic of livers affected by UC.

## 2. Materials and Methods

### 2.1. Animals

Male BALB/cKorl mice were obtained from the Department of Laboratory Animal Resources of the National Institute of Food and Drug Safety Evaluation (NIFDS, Cheongju, Korea) for the induction of colitis using dextran sodium sulfate (DSS). All animals were acclimated in temperature- (22 °C ± 2 °C) and humidity (55 ± 5%)-controlled rooms under a 12 h light/dark cycle for 7 days prior to use. Before starting any experimental procedure, animals of all experimental groups were weighed and gently manipulated in the laboratory environment for 30 min every day for at least 1 week, to minimize stress. The mice were allowed ad libitum access to standard laboratory chow diet and water until they reached the desired age (6–7 weeks) and/or weight (19–22 g). The animal protocol for this study was approved by the Institutional Animal Care and Use Committee of Pusan National University (Approval Number: PNU-2019-2229).

### 2.2. Induction of Ulcerative Colitis

Colitis was induced by feeding the mice for 7 days with control (*n* = 6), 2% (*n* = 6), 3.5% (*n* = 6), or 5% (*n* = 6) DSS (molecular weight, 40 kDa; ICN Biomedicals Inc, Cleveland, OH, USA) in their drinking water, and the DSS solution was replenished daily. Control mice received normal drinking water.

### 2.3. Assessment of the Disease Activity Index

The disease activity index (DAI) was determined by scoring the stool consistency, bleeding, and weight loss after colitis induction, according to the classical scoring system suggested by Cooper [28], as detailed in Table 1. All parameters were examined and scored from day 0 to day 7 during DSS treatment.

### 2.4. Examination of Alanine Aminotransferase (ALT) and Aspartate Aminotransferase (AST) Activities

Serum samples were obtained from the abdominal aorta of each test mouse using BD Microtainer Serum Collection Tubes (Becton, Dickinson and Company, Franklin Lakes, NJ, USA). The sera were stored at −80 °C until analysis. The serum activities of alanine aminotransferase (ALT) and aspartate aminotransferase (AST) were measured using the protocol described by Reitman and Frankel [29] and spectrophotometrically quantified using a Multiskan GO reader (Thermo Scientific, Waltham, MA, USA).

### 2.5. Histopathological Analysis

Colons were excised, and segments of the transverse colon were fixed in neutral-buffered formalin. Each colon tissue segment was washed in 0.1 M phosphate buffer (pH 7.4), and its length and weight were then measured. For histopathological analysis, small pieces of colon and the left lateral lobe of the liver were fixed in 10% phosphate-buffered formalin and embedded in low-melting-point paraffin. The tissue sections (5 µm) were stained with hematoxylin-eosin (HE) and examined by microscopy.

### 2.6. Sample Collection and Preparation

Metabolites were extracted from the right posterior lobe of the liver (0.25 mg) using distilled water, methanol, and chloroform. This extraction method is a modification of Bligh and Dyer’s method [30]. Briefly, 1.6 mL cold methanol and 0.6 mL distilled water were added to each liver sample. The samples were vortexed for 5 min and sonicated for 15 min; this was followed by the addition of 0.8 mL cold chloroform and keeping the samples in an ice bath for 10 min. Next, 0.8 mL cold chloroform and 0.8 mL distilled water were added. The final mixture was centrifuged at 1000× *g* for 10 min at 4 °C. The polar phase supernatant of each sample (2 mL) was transferred to a glass vial and lyophilized. After lysophilization, the samples were dissolved in 700 µL deuterium oxide (D_2_O, 99.9% D) containing 2 mM 3-(Trimethylsilyl) propionic-2,2,3,3-d_4_ acid sodium salt (TSP-d_4_) as the reference (0 ppm). All samples (700 µL) were then transferred to 5 mm NMR tubes for quantification.

### 2.7. NMR Analysis

All ^1^H-NMR spectra were acquired using a 600 MHz Agilent NMR spectrometer (Agilent, Santa Clara, CA, USA) operating at 600.167 MHz (14.1T). A Carr-Purcell-Meiboom-Gill (CPMG) pulse sequence was used to suppress the water and macromolecule peak. The ^1^H-NMR spectra were measured using a 9.8 µs 90° pulse, 1.5 s relaxation delay, 3 s acquisition time, and 13 min total acquisition time. A total of 128 scans were acquired for each sample at a spectral width of 24,038.5 Hz.

### 2.8. LC-MS/MS Analysis

After NMR analysis, each sample was reused. The samples were centrifuged at 10,000× *g* for 10 min at 4 °C, and the supernatant (500 µL) was diluted in water 1:1 (*v*/*v*). Chromatography was performed on an Agilent 1290 infinity UHPLC system (Agilent, Santa Clara, CA, USA) using a 2.1 × 100 mm (1.7 µm) HSS T3 Acquity (Waters, Milford, MA, USA). The mobile phase consisted of (A) 0.1% formic acid in water and (B) 0.1% formic acid in acetonitrile. The flow rate was 300 µL/min, and the injection volume was 3 µL for liver samples. A linear gradient with the following proportions (*v*/*v*) of phase B (t, %B) was used: (2, 1), (6, 15), (9, 50), (12, 95), (13, 1), and (16, 1). The UHPLC system was coupled with a quadrupole Time-of-Flight (Q-TOF) 4600 system (SCIEX, Framingham, MA, USA) and autosampler G4226A (Agilent). The autosampler temperature was set at 4 °C. TOF MS and MS/MS acquisition were performed in positive ionization and 50–1000 (*m*/*z*) scan modes.

For reproducibility of the HPLC-MS/MS analysis, quality control (QC) samples were analyzed once every seven samples to monitor the stability of the system. The QC sample consisted of 18 standard metabolites. The blank samples (50% acetonitrile) were run after seven injections, in order to monitor background noise. Additionally, an automated calibration was run after seven injections. The use of an automated calibrant delivery system, employing a calibration standard solution, ensured that the mass accuracy of the system was maintained throughout batch acquisition.

### 2.9. Metabolite Identification and Statistical Analysis

Chenomx NMR suite 8.4 (Chenomx Inc., Edmonton, AB, Canada), which includes an accurate library of fully searchable pH and magnetic field strength-dependent data, was used to enable an accurate qualitative and quantitative analysis of metabolite NMR data. Additionally, for more accurate quantification, single spectral and overlapping spectral areas were confirmed by spike-in experiments with authentic standards and 2D correlation spectroscopy (COSY) NMR spectra. The TSP-d_4_ peak at 0 ppm was used as the reference to calibrate the chemical shift. Each ^1^H-NMR spectrum was binned from 0.5 to 10 ppm, and the water peak area (4.7–4.9 ppm) was excluded. The binning size was 0.001 ppm, and the spectra were normalized to the total area. All binning data were imported into SIMCA-P+ 12.0 software (Umetrics, Umeå, Sweden). The multivariate analyses were performed using orthogonal partial least squares discriminant analysis (OPLS-DA), with unit variance scaling. In MS data analysis, to perform the library search and quantification of the metabolites, complete raw data were processed using the PeakView 2.2 software with the MasterView 1.1 package and Metabolomics library 1.0 (SCIEX) containing MS/MS spectra of 536 metabolites acquired at 16 different collision energies. The qualitative and quantitative analysis was performed based on the mass accuracy, isotopic pattern fit, MS/MS fragmentation pattern library, searching and retention time. The MasterView search parameters were set as follows: XIC intensities, above 200 count or signal to noise ratio (S/N) > 10; XIC width, 0.02 Da; peak integration, mono isotope. Library search parameters were set as follows: search algorithm, confirmation search; precursor mass tolerance, 0.4 Da; fragment mass accuracy, 5 ppm; polarity filter, applied; intensity factor, 5. Peak lists, representing a matrix of m/z_RT pairs for each sample, were extracted from the raw data, and the peak intensity of each sample was normalized to a total area sum using the MarkerView 1.3 software (SCIEX). The MarkerView search parameters were set as follows: RT range, 1–10 min; approximate LC peak width, 10 sec; minimum intensity in counts, 5; RT tolerance, 0.2 min; mass tolerance, 5 ppm; intensity threshold, 200; remove peaks in, <5 samples. For OPLS-DA analysis, the normalized data were imported into the SIMCA-P+ 12.0 software and were scaled through unit variance scaling.

## 3. Results

### 3.1. Induction of Colitis in DSS-Treated Mice

**Figure 1:**
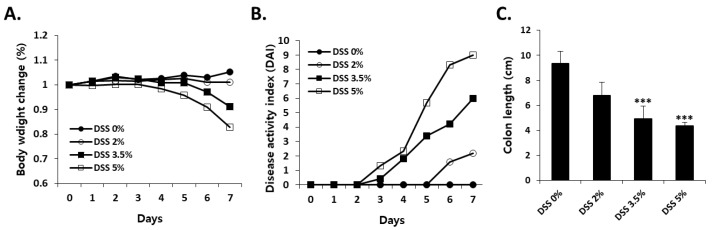
Assessment of (**A**) body weight change, (**B**) disease activity index (DAI), and (**C**) colon length in dextran sodium sulfate (DSS)-treated mice. Mice were given 2%, 3.5%, and 5% DSS in drinking water for 7 days. Each value is presented as the mean ± SD for six mice. *** Significantly different from the control (DSS 0%) (Student’s t-test, *p* < 0.001).

**Figure 2:**
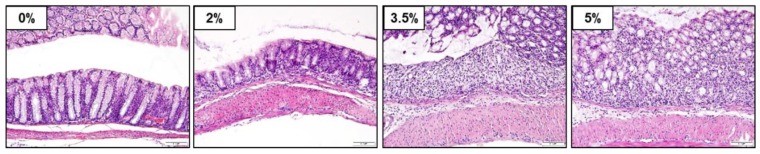
Histopathological analysis of the colon of the DSS-induced colitis mouse model. Colon tissues were stained with H&E (200× magnification).

To characterize the dose dependence of DSS-induced colitis, mice were administered 0% (control), 2%, 3.5%, or 5% DSS for 7 days. There was no significant difference in water intake (Figure S1A), but food intake significantly decreased, depending on the DSS concentration, from day 6 (Figure S1B). The time-course-dependent changes in the body weight, DAI, and colon length were then determined. Body weight started to decrease from day 4 in 5% DSS-treated mice. Significant differences in body weight were observed between the groups on day 7 in a dose-dependent manner (Figure 1A). The DAI, calculated based on the percentage of body weight decrease, diarrhea, and bloody feces, increased significantly from day 3 and 4 in the 5% and 3.5% DSS-treated groups, respectively (Figure 1B). The DAI of 2% DSS-treated mice increased significantly compared to that of the control group mice from day 6. The average colon lengths were 9.4, 6.8, 4.9, and 4.4 cm in control, 2%, 3.5%, and 5% DSS-treated mice, respectively (Figure 1C). Figure 2 shows the representative H&E-stained images of the colons on day 7 for mice treated with 2%, 3.5%, and 5% DSS and the untreated mice. Colons of the untreated mice had intact mucosa, whereas inflammatory cell infiltration was observed in the colons of the mice in 2%, 3.5%, and 5% DSS-treated groups. An increase in the thickness of the mucosa and submucosa and erosion of the epithelium were observed in the colons of 3.5% DSS-treated mice, while progressive alteration of the entire epithelium was detected in the colons of 5% DSS-treated mice.

### 3.2. Effect of DSS-Induced Colitis on Liver Injury Parameters

**Figure 3:**
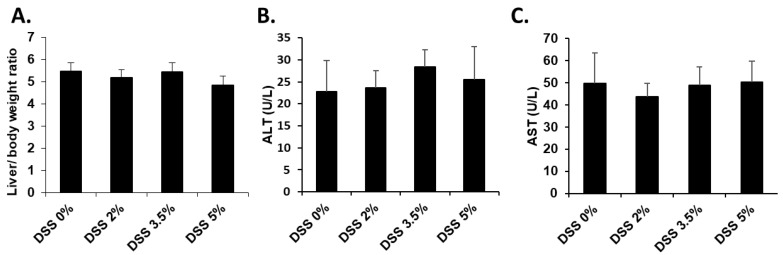
Changes in liver injury parameters. (**A**) Liver-to-body weight (**B**) and (**C**) serum activities of alanine aminotransferase (ALT) and aspartate aminotransferase (AST) in the DSS-treated mice.

**Figure 4:**
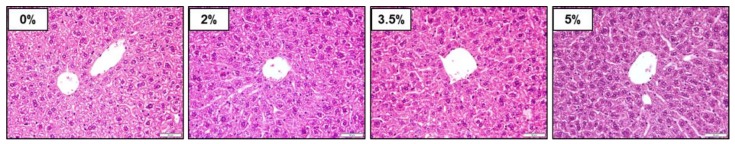
Histopathological changes in the liver of the DSS-induced colitis model. Liver tissues were stained with H&E (400× magnification).

To evaluate whether acute colitis induced by DSS treatment causes liver injury, an analysis of biochemical indexes of liver damage, such as the relative liver weight, serum activity of ALT and AST, and histology, was conducted on day 7 of DSS administration. No changes were observed in the liver-to-body weight ratio in either the DSS-treated group or the control group (Figure 3A). The serum activities of ALT and AST in the DSS-treated group were comparable to those in the control group (Figure 3B,C) and, in agreement with these results, neither morphological differences nor hepatocellular necrosis were observed in the DSS-treated groups (Figure 4).

### 3.3. Metabolomic Analysis of the Liver of DSS-Induced Colitis Mice

#### 3.3.1. NMR Data

**Figure 5:**
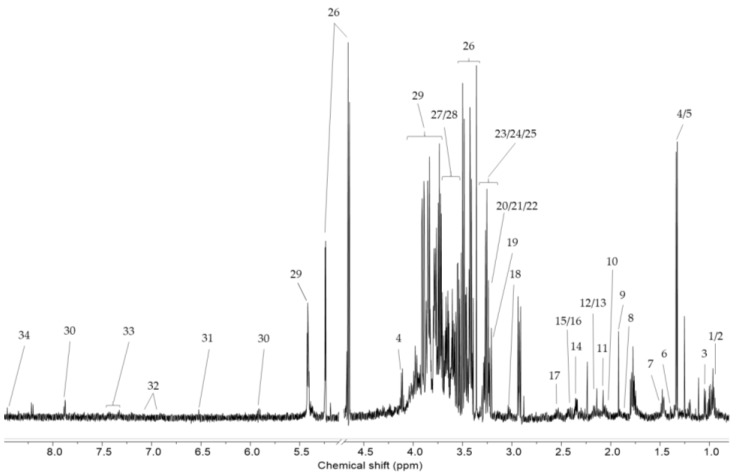
The representative 600 MHz ^1^H-NMR spectra of rat liver extracts. Key: 1, isoleucine; 2, leucine; 3, valine; 4, lactate; 5, threonine; 6, acetoin; 7, alanine; 8, arginine; 9, acetate; 10, proline; 11, N-acetylglucosamine; 12, methionine; 13, 2-octenoate; 14, glutamate; 15, pyroglutamate; 16, glutamine; 17, glutathione; 18, dimethylamine 19, lysine; 20, choline; 21, O-phosphocholine; 22, carnitine; 23, sn-glycerol-3-phosphocholine; 24, betaine; 25, taurine; 26, trimethylamine N-oxide; 27, glucose; 28, glycerol; 29, glycine; 30, maltose; 31, fumarate; 32; tyrosine; 33, phenylalanine; 34, formate.

**Figure 6:**
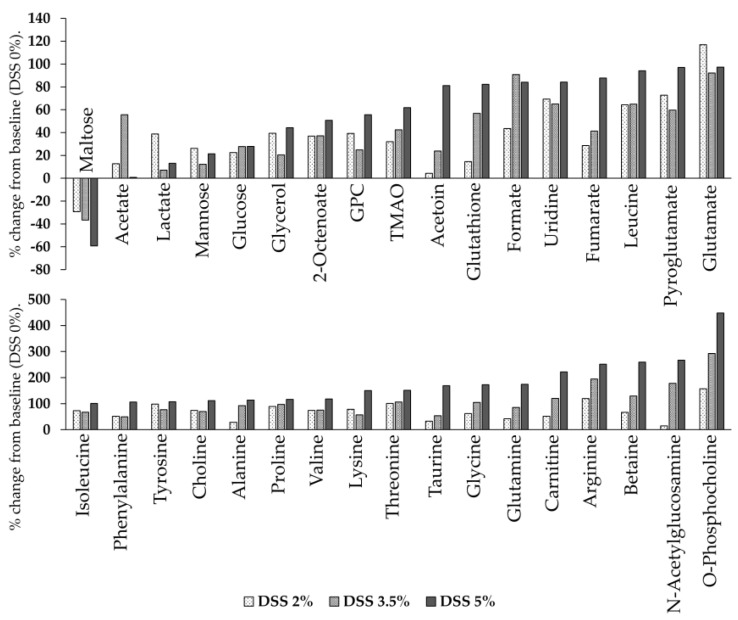
The percent change in metabolite levels in the DSS-treated mouse liver relative to the control mouse liver. % Change = ([DSS-treated liver]–[control liver])/[control liver] × 100). The concentrations of metabolites were calculated through the integration of peak areas using Chenomx. GPC, sn-Glycero-3-phosphocholine; TMAO, Trimethylamine N-oxide.

The ^1^H-NMR spectrum data were analyzed for liver samples of all mice in the control group (0% DSS-treated mice) and ulcerative colitis group (2% DSS-, 3.5% DSS-, and 5% DSS-treated mice). Figure 5 shows the representative ^1^H-NMR spectra for a mouse liver sample with assigned metabolites. For the liver samples, a total of 34 metabolites were quantified. The average concentration of the metabolites, as determined using Student’s t-test, is shown in Table 2. The levels of most metabolites, including those of amino acids and their derivatives (alanine, arginine, betaine, glutamate, glutamine, glutathione, glycine, isoleucine, leucine, lysine, phenylalanine, proline, pyroglutamate, threonine, tyrosine, and valine), lipids (2-octenoate, choline, glycerol, O-phosphocholine, and sn-glycero-3-phosphocholine), sulfur compounds (methionine and taurine), N-acetylglucosamine, carnitine, formate, fumarate, lactate, trimethylamine N-oxide, uridine, glucose, and mannose, increased, while the level of maltose decreased in the DSS-treated group compared to the control group. Therefore, the levels of 33 out of 34 metabolites increased, while the level of one metabolite decreased in the DSS-treated group compared to the control group. In particular, arginine, betaine, carnitine, N-acetylglucosamine, and O-phosphocholine levels were significantly increased with the increasing dose of DSS. Figure 6 shows the relative concentration of these metabolites in the DSS-treated group and control group livers.

#### 3.3.2. MS Data

**Figure 7:**
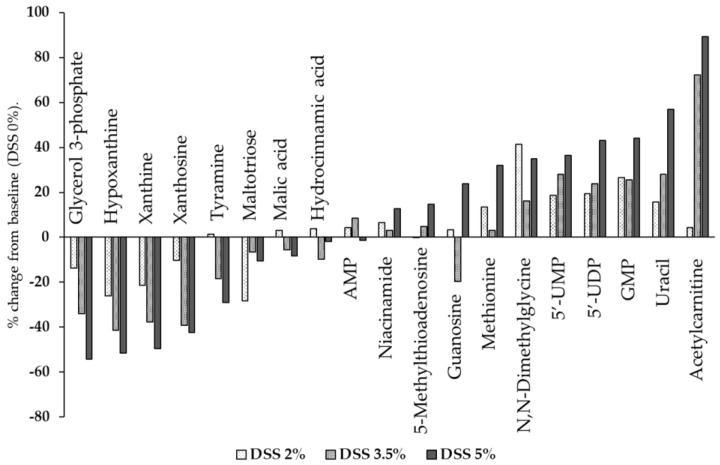
The percent change in liver metabolite levels in the DSS-treated mouse liver relative to the levels in normal liver. % Change = ([DSS-treated liver]–[control liver])/[control liver] × 100). The concentrations of metabolites were calculated based on the integration of peak areas using SCIEX peak view. AMP, adenosine monophosphate; 5′-UMP, uridine 5′-monophosphate; 5′-UDP, uridine 5′-diphosphate; GMP, guanosine monophosphate.

Mass spectrometry analysis identified changes in some metabolites that were not identified in the NMR, such as nucleic acids (5’-methylthioadenosine, adenosine monophosphate, guanosine, guanosine monophosphate, uridine 5’-diphosphate, uridine 5’-monophosphate, and xanthosin), purines (hypoxanthine and xanthine), pyrimidines (uracil), *N*,*N*-dimethylglycine, glycerol 3-phosphate, hydrocinnamic acid, acetylcarnitine, malic acid, maltoriose, methionine, niacinamide, and tyramine. The average concentrations of these metabolites, as determined using the Student’s t-test, are shown in Table 3, and Figure 7 shows the relative concentrations of these metabolites in the DSS-treated group liver compared to the control group liver. From among the abovementioned 19 metabolites, the levels of six metabolites increased (methionine, *N*,*N*-dimethylglycine, uridine 5’-monophosphate, uridine 5’-diphosphate, guanosine monophosphate, uracil, and acetylcarnitine), while those of five metabolites decreased (glycerol 3-phosphate, hypoxanthine, xanthine, xanthosine, and maltotriose) in the DSS-treated group compared to the control group. Overall, the levels of purine metabolites (hypoxanthine, xanthine, and xanthosine) decreased and those of pyrimidine metabolites (uridine 5’-monophosphate, uridine 5’-diphosphate, uridine, and uracil) increased in the DSS-treated group compared to the control group.

### 3.4. Multivariate Statistical Analysis

#### 3.4.1. NMR Data

**Figure 8:**
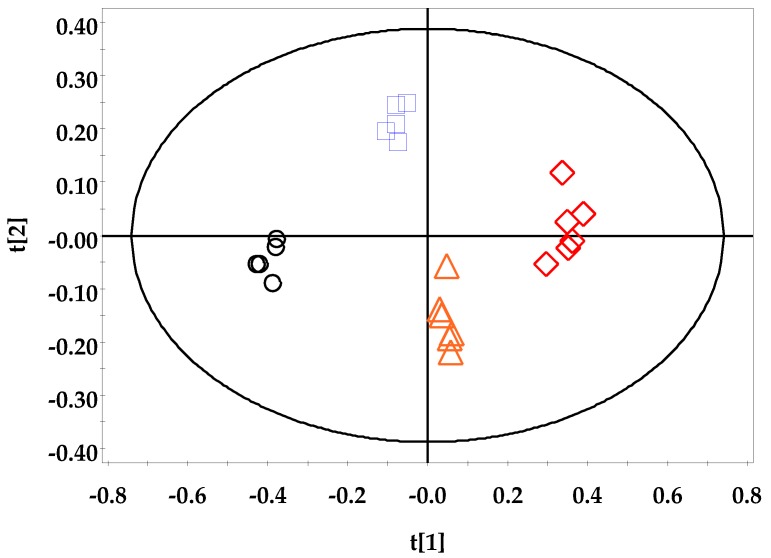
OPLS-DA score plot based on the ^1^H-NMR spectra of the DSS-treated group and control group. ○: DSS 0%; □: DSS 2%; △: DSS 3.5%; ◇: DSS 5% (*R*^2^*X* = 0.738, *R*^2^*Y* = 0.919, and *Q*^2^ = 0.258).

To investigate the ^1^H-NMR spectrum data and remove the possible intragroup confounding factors or structure noise, OPLS-DA was applied. The OPLS-DA results are shown in Figure 8. The OPLS-DA score plots (*R*^2^*X* = 0.738, *R*^2^*Y* = 0.919, and *Q*^2^ = 0.258) demonstrated significant differences between the DSS-treated groups and the control group. The *R*^2^ value is defined as the proportion of variance in the data explained by the models and the overall goodness of fit, and the *Q*^2^ value is defined as the proportion of variance in the data predictable by the model and the overall cross validation coefficient.

#### 3.4.2. UPLC-QTOF-MS/MS Data

**Figure 9:**
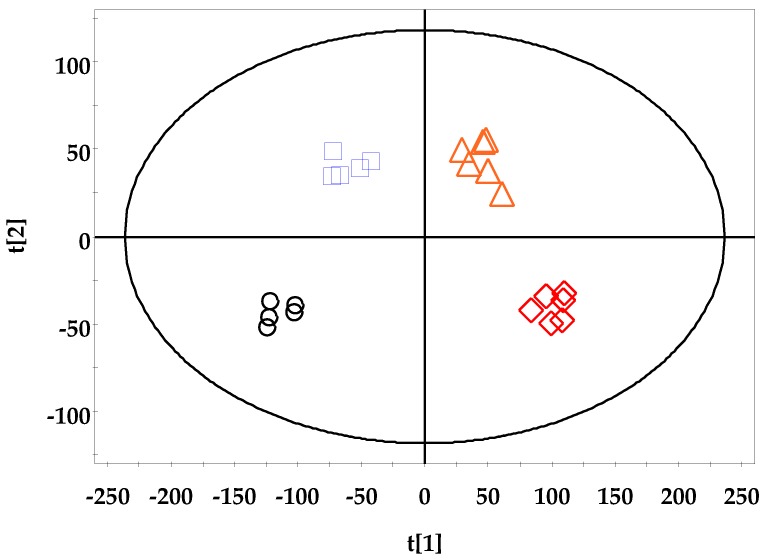
OPLS-DA score plot based on the MS spectra of the DSS-treated group and control group. ○: DSS 0%; □: DSS 2%; △: DSS 3.5%; ◇: DSS 5% (*R*^2^*X* = 0.69, *R*^2^*Y* = 0.97, and *Q*^2^ = 0.554).

The OPLS-DA results are shown in Figure 9. The OPLS-DA score plots (*R*^2^*X* = 0.69, *R*^2^*Y* = 0.97, and *Q*^2^ = 0.554) demonstrated a differentiation between the DSS-treated groups and the control group, depending on the concentration of DSS used. The high DSS concentration groups (DSS 3.5% and DSS 5%) and low DSS concentration groups (DSS 0% and DSS 2.5%) are separated on the x = 0 axis.

## 4. Discussion

Recent experimental and clinical studies have revealed the pivotal role of the gut–liver axis in the onset of metabolic disturbances related to lipid and glucose homeostasis [31,32,33,34,35]. In particular, the resident microbiota in the gut have been recognized as key players in the gut–liver interaction. They not only influence the absorption and disposal of nutrients in the liver, but also condition hepatic inflammation by supplying toll-like receptor ligands, which can stimulate the liver cells to produce pro-inflammatory cytokines. The gut–liver crosstalk is implicated in promoting simple lipid accumulation, as well as in the initiation of inflammation and fibrogenesis in the liver. Reportedly, NAFLD and primary sclerosing cholangitis have been found to occur in patients with IBD [6,7,8,36]. Therefore, the modification of intestinal bacterial flora by specific probiotics has been proposed as a therapeutic approach for the treatment of a wide spectrum of fatty liver disease. In spite of all the studies mentioned above, communication between the gut and liver still requires further exploration.

In order to identify the direct link between liver metabolism and the intestinal barrier function, we examined various metabolites in the liver of mice with DSS-induced acute colitis, which is a well-established mouse model of UC and has features similar to human UC [37,38]. In particular, increasing dosages of DSS from 0% to 5% were administered to the mice to study different grades of colitis. The supplementation of DSS induced significant colon injury, with inflammatory cell infiltration and disruption of the epithelial barrier, in a dose-dependent manner, as reported previously [39]; however, the liver was not damaged, as evidenced by the results of the analysis of ALT and AST serum activity, as well as histology. Although liver injury was not induced, in a recent study, we noted that the combined administration of a high-fat diet and DSS to C57BL/6 mice not only aggravated lipid accumulation, but also induced steatohepatitis in the liver [40]. These results suggest that changes in the intestinal environment induced by DSS could affect hepatic metabolism and exacerbate the metabolic burden of high-fat diets in the liver.

Endogenous metabolites are known to be easily changed by environmental stress, chemical exposure, and genetic variations, even in the absence of a diseased state; thus, metabolite analysis provides comprehensive information on energy metabolism, precursors of proteins and carbohydrates, gene expression regulation, and signaling molecules [41,42,43,44,45,46]. In this study, we analyzed small molecular metabolites, including the intermediate and final products of cellular regulatory processes, and they could be clearly distinguished by multivariate statistical analysis (Figure 8 and Figure 9), depending on the supplemented DSS dosage.

**Figure 10:**
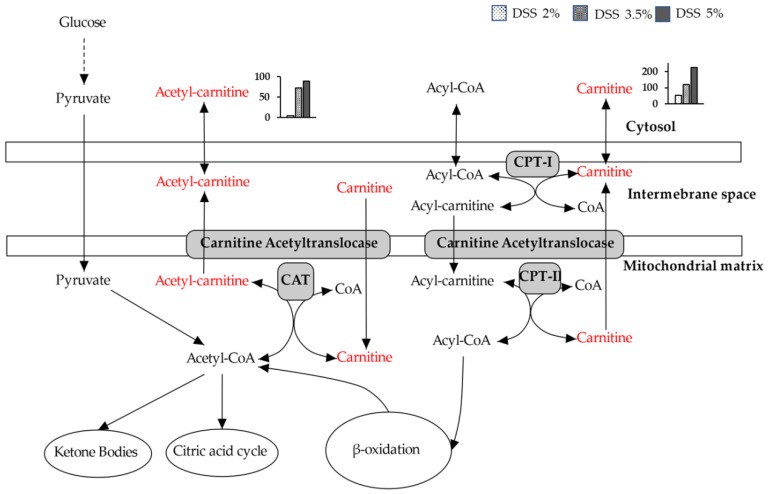
Overview of acetyl-carnitine- and carnitine-related metabolism in the liver of mice with ulcerative colitis (UC).

The metabolites determined by ^1^H-NMR and UPLC-QTOF-MS/MS in the present study responded differently to different concentrations of DSS and significant differences (*p* < 0.05) in their levels were observed between the DSS-treated group and control group. The levels of acetyl-carnitine and carnitine were elevated in the DSS-treated group. Acetyl-carnitine and carnitine are important for the β-oxidation of fatty acids, which mainly occurs in the mitochondria (Figure 10), to regulate lipid metabolism and transport fatty acids into the mitochondria [47,48]. They also have antioxidant and anti-inflammatory properties [49]. Carnitine also has an antioxidant and anti-inflammatory capacity [48,50]. Some studies have reported that acetyl-carnitine and carnitine are associated with liver function, liver diseases, neurologic disorders, and IBD [22,51,52,53,54]. Therefore, liver damage due to intestinal inflammation occurs as a result of changes in the concentrations of acetyl-carnitine and carnitine. These changes suggest that intestinal inflammation affects oxidative stress, inflammatory cells, osmosis, and β-oxidation of fatty acids in the liver.

**Figure 11:**
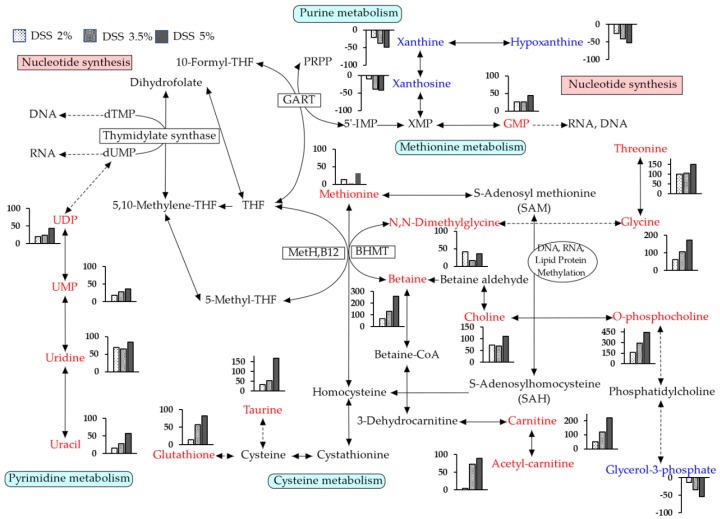
Summary of one-carbon metabolism, purine metabolism, pyrimidine metabolism, and transsulfuration pathways in the liver with UC.

The levels of methionine, choline, betaine, *N*,*N*-dimethylglycine, glycine, threonine, glutathione, and taurine, which are related to the pathway of one-carbon metabolism, were significantly increased in the DSS-treated group. One-carbon metabolism plays an important role in the transsulfuration pathway and nucleotide synthesis, which is essential for DNA replication and repair [55]. Moreover, it transfers the methyl group to acceptors, including DNA, RNA, proteins, and phospholipids [56]. Figure 11 shows a summary of one-carbon metabolism, purine metabolism, pyrimidine metabolism, and the transsulfuration pathway in the liver with UC. The levels of choline and O-phosphocholine, which are essential nutrients, and phospholipids in the mitochondrial membranes [57,58], were increased. These are associated with one-carbon metabolism via betaine and lipid metabolism. The changes in choline and O-phosphocholine levels suggested the possibility of damage to the membrane or progress towards membrane disruption [59]. The intestine in the DSS-treated group is in a high osmotic environment, which causes osmotic stress, cell cycle arrest, DNA damage, oxidative stress, and inflammation [60]. In our study, one-carbon metabolism-related changes in the metabolite levels occurred in response to the severity of intestinal inflammation. Intestinal inflammation may affect epigenetic regulation. Moreover, pyrimidine metabolism-related metabolite (uracil, uridine, UMP, and UDP) levels and purine metabolism-related metabolite (xanthosine, xanthine, hypoxanthine, and GMP) levels were altered. These metabolites are associated with the synthesis of nucleotides and are known to act against inflammation and DNA damage. The increase in the levels of betaine, carnitine, and proline, which are osmoprotectants, may help reduce osmotic stress [48,61,62]. Betaine can be metabolized to carnitine via a multistep process. Therefore, changes in betaine levels may have affected the carnitine levels. Betaine can be utilized in different metabolic pathways, depending on the present environmental stressors, such as osmotic stress, oxidative stress, inflammation, and energy metabolism disorders [63]. In the liver, the primary role of betaine is as a methyl group donor [64,65]. The level of betaine is significantly elevated in the DSS-treated group, suggesting that intestinal inflammation leads to the upregulation of one-carbon metabolism, which plays an important role in maintenance of the inflammatory response. Considering acute intestinal injury of this model, it seems that the altered metabolites in one-carbon, purine, pyrimidine, and transsulfuration pathways may be compensatory or acute phase responses to protect the liver against changes in the intestinal environment.

In summary, the results of the present study show that the overall changes in liver metabolism in response to DSS administration are clearly associated with the severity of colon injury and thus suggest the importance of gut condition in the maintenance of liver homeostasis. However, the significance of metabolic changes in response to DSS-induced acute colitis remains unknown. Further investigation of the role of colitis-specific metabolites in the liver may prove to be useful for clinical application and preventive approaches against colitis-associated liver disease.

## Figures and Tables

**Table 1:** Disease activity index score parameters.

Weight Loss	Stool Consistency	Bleeding
0 = no weight loss	0 = formed	0 = normal color stool
1 = 5–10% weight loss	1 = mild-soft	1 = brown color stool
2 = 11–15% weight loss	2 = very soft	2 = reddish color stool
3 = 16–20% weight loss	3 = watery stool	3 = bloody stool
4 = 20% weight loss		

**Table 2:** The relative concentrations of metabolites acquired by ^1^H-NMR spectroscopy from DSS 0%, DSS 2%, DSS 3.5%, and DSS 5% livers.

	DSS 0%	DSS 2%		DSS 3.5%		DSS 5%	
	Mean ± SE	Mean ± SE	(*p*-value)	Mean ± SE	(*p*-value)	Mean ± SE	(*p*-value)
2-Octenoate	1.961 ± 0.236	2.686 ± 0.641	(0.1598)	2.690 ± 0.359	(0.0701)	2.956 ± 0.405	(0.0378) *
Acetate	2.465 ± 0.224	2.779 ± 0.272	(0.1993)	3.835 ± 0.489	(0.0208) *	2.485 ± 0.368	(0.4825)
Acetoin	1.256 ± 0.494	1.311 ± 0.332	(0.4648)	1.556 ± 0.198	(0.2807)	2.277 ± 0.548	(0.1039)
Alanine	1.141 ± 0.178	1.469 ± 0.121	(0.0828)	2.192 ± 0.455	(0.0390) *	2.439 ± 0.511	(0.0272) *
Arginine	0.632 ± 0.107	1.388 ± 0.295	(0.0214) *	1.862 ± 0.452	(0.0194) *	2.220 ± 0.420	(0.0043) **
Betaine	0.597 ± 0.136	0.996 ± 0.177	(0.0559)	1.369 ± 0.323	(0.0357) *	2.147 ± 0.566	(0.0190) *
Carnitine	0.631 ± 0.042	0.958 ± 0.180	(0.0575)	1.391 ± 0.285	(0.0201) *	2.034 ± 0.627	(0.0369) *
Choline	1.269 ± 0.362	2.208 ± 0.466	(0.0749)	2.154 ± 0.401	(0.0712)	2.684 ± 0.350	(0.0104) *
Formate	1.400 ± 0.307	2.010 ± 0.452	(0.1485)	2.671 ± 0.329	(0.0107) *	2.578 ± 0.455	(0.0353) *
Fumarate	1.632 ± 0.237	2.100 ± 0.341	(0.1461)	2.307 ± 0.339	(0.0762)	3.066 ± 0.522	(0.0223) *
Glucose	4.696 ± 0.216	5.754 ± 0.618	(0.0723)	6.002 ± 0.372	(0.0092) **	6.004 ± 0.269	(0.0025) **
Glutamate	1.365 ± 0.199	2.961 ± 0.575	(0.0153) *	2.623 ± 0.287	(0.0036) **	2.693 ± 0.337	(0.0053) **
Glutamine	0.967 ± 0.167	1.374 ± 0.286	(0.1274)	1.793 ± 0.491	(0.0884)	2.656 ± 0.309	(0.0007) ***
Glutathione	1.522 ± 0.086	1.744 ± 0.235	(0.2004)	2.388 ± 0.647	(0.1299)	2.774 ± 0.238	(0.0007) ***
Glycerol	2.936 ± 0.200	4.093 ± 0.689	(0.0728)	3.536 ± 0.217	(0.0380) *	4.235 ± 0.352	(0.0071) **
Glycine	0.957 ± 0.073	1.550 ± 0.178	(0.0075) **	1.961 ± 0.316	(0.0099) **	2.612 ± 0.557	(0.0128) *
Isoleucine	1.323 ± 0.051	2.287 ± 0.495	(0.0443) *	2.212 ± 0.327	(0.0187) *	2.658 ± 0.508	(0.0211) *
Lactate	3.359 ± 0.289	4.661 ± 0.524	(0.0306) *	3.600 ± 0.293	(0.2884)	3.797 ± 0.461	(0.2317)
Leucine	1.612 ± 0.074	2.648 ± 0.437	(0.0238)*	2.659 ± 0.366	(0.0156)*	3.130 ± 0.466	(0.0085) **
Lysine	0.924 ± 0.089	1.647 ± 0.307	(0.0269) *	1.448 ± 0.304	(0.0821)	2.310 ± 0.598	(0.0335) *
Maltose	2.686 ± 0.266	1.898 ± 0.426	(0.0775)	1.704 ± 0.482	(0.0636)	1.096 ± 0.257	(0.0010) **
Mannose	4.006 ± 0.089	5.056 ± 0.812	(0.1174)	4.499 ± 0.280	(0.0793)	4.866 ± 0.259	(0.0090) **
N-Acetylglucosamine	0.515 ± 0.030	0.591 ± 0.160	(0.3264)	1.431 ± 0.354	(0.0221) *	1.889 ± 0.559	(0.0267)*
O-Phosphocholine	0.319 ± 0.041	0.820 ± 0.263	(0.0484) *	1.251 ± 0.311	(0.0122) *	1.746 ± 0.595	(0.0292) *
Phenylalanine	1.249 ± 0.115	1.893 ± 0.359	(0.0631)	1.866 ± 0.227	(0.0245) *	2.580 ± 0.625	(0.0447) *
Proline	1.295 ± 0.143	2.452 ± 0.469	(0.0230) *	2.550 ± 0.408	(0.0127) *	2.801 ± 0.373	(0.0034) **
Pyroglutamate	1.194 ± 0.120	2.062 ± 0.517	(0.0704)	1.907 ± 0.253	(0.0209) *	2.353 ± 0.565	(0.0505)
Taurine	0.775 ± 0.066	1.031 ± 0.148	(0.0763)	1.191 ± 0.229	(0.0720)	2.084 ± 0.668	(0.0556)
Threonine	0.895 ± 0.113	1.799 ± 0.504	(0.0592)	1.847 ± 0.325	(0.0157) *	2.247 ± 0.493	(0.0187) *
Trimethylamine N-oxide	2.271 ± 0.130	2.998 ± 0.537	(0.1124)	3.234 ± 0.217	(0.0028) **	3.675 ± 0.513	(0.0192) *
Tyrosine	1.355 ± 0.160	2.687 ± 0.609	(0.0337) *	2.394 ± 0.265	(0.0055) **	2.810 ± 0.373	(0.0044) **
Uridine	1.593 ± 0.155	2.697 ± 0.498	(0.0336) *	2.627 ± 0.418	(0.0303) *	2.934 ± 0.399	(0.0088) **
Valine	1.186 ± 0.073	2.066 ± 0.390	(0.0287)*	2.075 ± 0.322	(0.0182) *	2.586 ± 0.557	(0.0250) *
sn-Glycero-3-phosphocholine	2.233 ± 0.224	3.111 ± 0.674	(0.1258)	2.788 ± 0.302	(0.0946)	3.474 ± 0.351	(0.0097) **

Metabolites that were significantly different between the different DSS-treated groups are shown in bold and with an asterisk. * *p* < 0.05; ** *p* < 0.01; *** *p* < 0.001, compared to the control (DSS 0%).

**Table 3:** The relative concentrations of marker metabolites, acquired by MS spectroscopy of DSS 0%, DSS 2%, DSS 3.5%, and DSS 5% livers.

	DSS 0%	DSS 2%		DSS 3.5%		DSS 5%	
	Mean ± SE	Mean ± SE	(*p*-value)	Mean ± SE	(*p* –value)	Mean ± SE	(*p* –value)
5’-Methylthioadenosine	5.973 ± 0.460	5.968 ± 0.519	(0.4974)	6.255 ± 0.472	(0.3410)	6.854 ± 0.242	(0.0538)
Adenosine monophosphate	5.340 ± 0.374	5.571 ± 0.318	(0.3249)	5.801 ± 0.482	(0.2418)	5.265 ± 0.522	(0.4571)
*N*,*N*-Dimethylglycine	2.866 ± 0.399	4.049 ± 0.412	(0.0365) *	3.332 ± 0.393	(0.2149)	3.870 ± 0.421	(0.0610)
Glycerol 3-phosphate	3.768 ± 0.278	3.248 ± 0.299	(0.1195)	2.486 ± 0.331	(0.0090) **	1.726 ± 0.204	(0.0001) ***
Guanosine	3.553 ± 0.513	3.673 ± 0.237	(0.4183)	2.853 ± 0.414	(0.1551)	4.398 ± 0.276	(0.0809)
Guanosine monophosphate	2.957 ± 0.231	3.740 ± 0.397	(0.0634)	3.714 ± 0.393	(0.0754)	4.258 ± 0.500	(0.0276)*
Hydrocinnamic acid	5.097 ± 0.582	5.289 ± 0.451	(0.3999)	4.601 ± 0.390	(0.2422)	5.007 ± 0.374	(0.4480)
Acetylcarnitine	1.652 ± 0.155	1.723 ± 0.192	(0.3905)	2.845 ± 0.307	(0.0050) **	3.129 ± 0.500	(0.0146) *
Malic acid	5.843± 0.250	6.029 ± 0.216	(0.2942)	5.513 ± 0.615	(0.3279)	5.356 ± 0.445	(0.1961)
Methionine	4.001 ± 0.281	4.535 ± 0.320	(0.1227)	4.120 ± 0.500	(0.4243)	5.282 ± 0.370	(0.0130)*
Maltotriose	4.091 ± 0.379	2.936 ± 0.093	(0.0091) **	3.828 ± 0.525	(0.3526)	3.666 ± 0.446	(0.2479)
Niacinamide	6.631 ± 0.628	7.072 ± 0.350	(0.2788)	6.837 ± 0.307	(0.3811)	7.468 ± 0.434	(0.1445)
Tyramine	2.660 ± 0.578	2.699 ± 0.623	(0.4825)	2.173 ± 0.313	(0.2283)	1.886 ± 0.186	(0.1004)
Uracil	3.633 ± 0.368	4.199 ± 0.201	(0.1070)	4.656 ± 0.360	(0.0400) *	5.697 ± 0.171	(0.0002) ***
Uridine 5’-diphosphate	3.030 ± 0.114	3.616 ± 0.176	(0.0118) *	3.751 ± 0.589	(0.1513)	4.340 ± 0.407	(0.0097)**
Uridine 5’-monophosphate	3.482 ± 0.173	4.130 ± 0.244	(0.0312) *	4.457 ± 0.445	(0.0458) *	4.750 ± 0.532	(0.0335) *
Xanthosine	3.480 ± 0.307	3.123 ± 0.198	(0.1784)	2.118 ± 0.460	(0.0216) *	2.010 ± 0.329	(0.0053) **
Hypoxanthine	4.463 ± 0.173	3.299 ± 0.179	(0.0008) ***	2.621 ± 0.237	(0.0001) ***	2.164 ± 0.257	(0.0000) ***
Xanthine	4.616 ± 0.226	3.635 ± 0.177	(0.0045) **	2.883 ± 0.242	(0.0003) ***	2.335 ± 0.241	(0.0000) ***

Metabolites that had significantly different concentrations between the different DSS-treated groups are shown in bold and accompanied by an asterisk. * *p* < 0.05; ** *p* < 0.01; *** *p* < 0.001, compared to the control (DSS 0%).

## Supplementary Materials

The following are available online at https://www.mdpi.com/2073-4409/9/2/341/s1: Figure S1: (A) Food intake and (B) water intake in DSS-treated mice. Mice were given 2%, 3.5%, and 5% DSS in drinking water for 7 days. Each value is represented as the mean ± SD of six mice.
