# Supplementary material for: Metabolomic Analysis of the Liver of a Dextran Sodium Sulfate-Induced Acute Colitis Mouse Model: Implications of the Gut–Liver Connection
Source: Cells. 2020 Feb 1;9(2):341. doi: 10.3390/cells9020341 (PMC7072179; doi:10.3390/cells9020341)
Supplement: Supplementary file 1 [file cells-09-00341-s001.pdf]

cells

MDPI

Supplementary figure

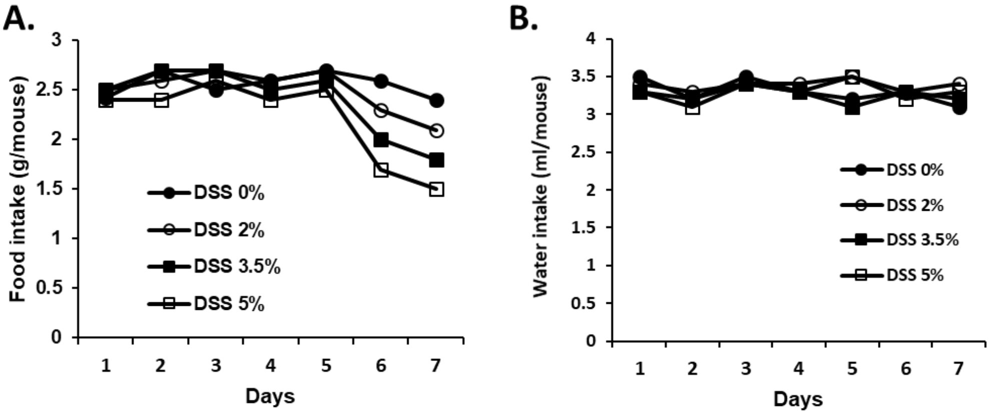

Figure S1. (A) Food intake and (B) water intake in DSS-treated mice. Mice were given 2, 3.5, 5% DSS in drinking water for 7 days. Each value is represented as mean ± SD of 6 mice.
